# Primary Care Career Perceptions: Comparing Temperament and Character Inventory Profiles of Medical Students with General Practitioners

**DOI:** 10.3390/ijerph23050658

**Published:** 2026-05-15

**Authors:** Anna Nánási, Viktor Rekenyi, Csongor István Szepesi, Eszter Kovács, Nóra Horváth, Armand Kun, László Róbert Kolozsvári

**Affiliations:** 1 Doctoral School of Health Sciences, University of Debrecen, 4032 Debrecen, Hungary; 2 Department of Family and Occupational Medicine, Faculty of Medicine, University of Debrecen, 4032 Debrecen, Hungary; 3 Department of Psychiatry, Faculty of Medicine, University of Debrecen, 4032 Debrecen, Hungary

**Keywords:** general practitioner shortage, career choice, TCI-55, medical students, primary care, Hungarian healthcare

## Abstract

**Highlights:**

**Public health relevance—How does this work relate to a public health issue?**

- The systemic shortage of General Practitioners (GPs) directly compromises the foundational tier of healthcare, threatening proactive disease prevention, chronic disease management, and overall population health outcomes on a global scale.
- Growing workforce deficits leave vulnerable populations without continuous primary care, forcing a shift from personalized preventive medicine to reactive, fragmented acute care, which exacerbates health inequities and overwhelms emergency infrastructures.

**Public health significance—Why is this work of significance to public health?**

- The critical unpopularity of family medicine among medical trainees represents a severe public health vulnerability, as a shrinking primary care workforce directly diminishes a national health system’s capacity to deliver equitable, community-level care.
- The profound disconnect between students’ unfounded negative stereotypes and the systemic burnout (e.g., heavy workloads, severe time constraints) experienced by practicing GPs fuels a vicious cycle that accelerates workforce attrition and threatens the quality and safety of patient care.

**Public health implications—What are the key implications or messages for practitioners, policy makers and/or researchers in public health?**

- Integrating structured temperament assessments into medical education serves as a strategic public health intervention, enabling policymakers and educators to purposefully identify and support trainees intrinsically suited for the rigors of primary care, thereby improving long-term workforce retention.
- Public health strategies must prioritize the occupational well-being of physicians by mitigating structural and administrative stressors, recognizing that a resilient, sustainable GP workforce is a fundamental prerequisite for effective population health management.

**Abstract:**

The structural integrity of the Hungarian healthcare system is threatened by a systemic shortage of General Practitioners (GPs), with 1021 districts remaining vacant as of March 2026. This study assessed demographic characteristics, perceived career attributes, and temperament and character traits of 144 medical students and 72 practicing GPs, achieving an overall response rate of 91.5%, using the TCI-55 inventory to identify factors influencing professional pathways. Statistical analysis utilized Mann–Whitney U, Chi-square, and Fisher’s exact tests. Results showed only 6 students indicated family medicine as their first choice, compared to 49 GPs (*p* < 0.001). Students frequently perceived the field as “boring” (82 students vs. 1 GP, *p* < 0.001) and burdened by “trivial patient problems” (44 students vs. 7 GPs, *p* = 0.001), while GPs highlighted “stress” (15 GPs vs. 4 students, *p* < 0.001) as a primary disadvantage. Students scored significantly higher in the temperament dimensions of Novelty Seeking (*p* < 0.001) and Reward Dependence (*p* < 0.001). Addressing these educational misperceptions and urgently mitigating the structural drivers of occupational stress and workforce fatigue are critical public health priorities required to ensure the sustainability of primary care, maintain the continuity of preventive services, and safeguard equitable population health outcomes.

## 1. Introduction

To understand the current challenges within Hungarian primary care, it is essential to frame them within the broader structural context of the national healthcare system. While Hungary maintains a historically high number of hospital beds—a hallmark of its “post-socialist legacy” and a traditionally hospital-centric model—the structural integrity of its healthcare system is increasingly threatened by a severe and systemic shortage of General Practitioners (GPs). In this system, GPs act as the foundational “gatekeepers”; patients generally must consult their GP to access state-funded specialized care, hospital admissions, or subsidies for essential medications. Therefore, a shortage of GPs is not merely an administrative issue, but a profound public health crisis [1].

This crisis is demonstrably worsening. As of October 2024, nearly 13% of GP districts—a total of 821 positions—were chronically vacant for over six months. By March 2026, this deficit had rapidly deteriorated, with over 16% of GP districts (1021 positions) unfilled. With 929 of these vacancies lasting longer than six months, roughly one million citizens are currently left without a permanent family doctor. This deficit creates a dangerous ripple effect: substitute doctors are forced to manage multiple vacant districts simultaneously alongside their own. This substitution doubles their workload and severely compromises the quality of care for an additional one million people. Consequently, primary care has transformed into a major bottleneck where physical infrastructure may exist, but the human capacity to provide essential services is rapidly evaporating [1,2].

Maintaining a sustainable healthcare system requires an adequate workforce of qualified professionals, which poses a significant challenge in Hungary, just as it does globally. An aging population, growing healthcare demands, and the emigration of medical professionals all contribute to the increasing strain on the national healthcare system. With approximately one-third of licensed doctors in Hungary already of retirement age, this situation is projected to deteriorate further in the coming years [2,3].

To address these systemic challenges, the Hungarian government introduced the ‘four-pillar model,’ a national policy framework designed to strengthen and renew primary care. This framework identifies four core areas of intervention: improving care organization, reforming financing, promoting the general practitioner (GP) career at an early stage, and embedding a stronger GP focus within medical education [3,4]. Within the context of our research, this model underscores that early educational interventions and career promotion are officially recognized as vital to resolving the workforce crisis. Conversely, despite these policy goals, the actual number of GP specializations issued annually in Hungary has not reached 150 since 2014. Considering the more than 6000 GP practices and an average service span of 40 years per physician, it is evident that the current vocational training system fails to provide adequate replacements. Given the rapidly aging demographic of GPs, the annual number of new specialists remains critically insufficient [5].

To halt these unfavorable trends and increase the popularity of the general practitioner career, it is crucial to map medical students’ perceptions and knowledge regarding their choice of specialty, with a particular focus on the GP profession.

Currently, the exact factors influencing this career choice are not fully understood. Previous studies have primarily examined the potential role of several non-modifiable factors, such as place of origin, gender, personality traits, and family background [6,7]. Furthermore, the perceived overall appeal of a specific medical field, along with quality-of-life factors such as work–life balance and family-friendly conditions, are increasingly recognized as having a significant influence on students’ career decisions [8,9]. Finally, the role of a student’s personality is increasingly emphasized. In this context, personality is defined as an individual’s relatively stable and enduring disposition, which manifests through consistent patterns of thought, emotion, and behavior [10,11].

Rather than assessing the broader concept of personality, it may be particularly valuable to specifically evaluate temperament when examining medical students’ career choices. Biopsychological models draw a sharp distinction between innate, genetically coded temperament and character acquired through learning. While character traits (such as cooperativeness or self-directedness) are continuously shaped by the sociocultural environment, university expectations, and hospital hierarchies, temperament traits (such as harm avoidance or reward dependence) remain biologically stable. They strongly influence an individual’s deep, instinctive emotional responses to stress, interpersonal dynamics, and clinical uncertainty. Because general practice inherently involves managing a broad spectrum of undifferentiated patient problems, fostering long-term doctor-patient relationships, and navigating high levels of diagnostic uncertainty, an early assessment of a student’s genetically stable temperament may offer a highly stable and enduring indicator of their long-term career fit. Because this approach is less susceptible to the socializing biases of medical training, it can help identify whether a student possesses the intrinsic disposition to thrive in primary care over the long term without burning out [12,13,14].

Because current admission and selection procedures fail to reliably or comprehensively identify the personality traits most desirable for specific medical disciplines, assessing students’ temperaments could serve as a vital tool to address this issue. The early identification of these traits not only enhances career counseling and guidance but also allows medical schools to tailor their curricula effectively. Research highlights that medical students intending to pursue rural or shortage specialties possess a distinct temperament profile, such as significantly lower Harm Avoidance and higher Self-Directedness. These intrinsic characteristics enable them to demonstrate greater adaptability and decision-making confidence, even when faced with high uncertainty, limited resources, and professional isolation. Therefore, a structured assessment of temperament is essential to purposefully train and counsel students who are naturally best equipped to handle these specific challenges, ultimately increasing the likelihood of their long-term retention in the rural workforce [14].

Assessing temperament is crucial not only for addressing the rural workforce challenge. Recent research utilizing the Temperament and Character Inventory (TCI) has revealed that specific temperament traits play a decisive role in medical specialty selection, often existing prior to residency training. The study suggests that students do not simply “acquire” these traits during their education but rather choose paths that align with their inherent psychological makeup. A key finding involves primary care specialties, where students scored significantly higher in Reward Dependence (RD). This indicates that those entering primary care possess a temperament characterized by higher sentimentality and a greater need for interpersonal connection, making them naturally suited for fields that require sustained patient rapport [13].

From a broader public health perspective, the critical shortage of general practitioners transcends mere occupational and administrative challenges; it directly undermines population health outcomes and exacerbates systemic health inequities. Primary care serves as the foundational pillar of any resilient healthcare system, playing an indispensable role in preventive medicine, early diagnosis, health promotion, and the continuous management of chronic non-communicable diseases [15]. Extensive epidemiological evidence consistently demonstrates that a robust primary care workforce—and continuity of care—is significantly associated with lower all-cause mortality, increased life expectancy, and a substantial reduction in avoidable hospital admissions and emergency department overutilization [16]. When large segments of the population lose continuous access to a dedicated family physician, the healthcare paradigm inevitably shifts from proactive, personalized prevention to reactive, fragmented acute care. This shift not only inflates overall systemic healthcare costs but also disproportionately harms vulnerable, aging, and rural populations, thereby widening the health disparity gap [17]. Our study aimed to assess the demographic characteristics and temperament traits of Hungarian medical students and general practitioners to determine whether a significant correlation exists between these factors and their career choices. By analyzing the psychological profiles of both future and practicing physicians, we sought to identify specific personality patterns that may influence the decision to pursue a medical career or a particular specialization. Ultimately, this research provides insight into how innate traits and background factors shape professional pathways within the Hungarian healthcare system.

## 2. Materials and Methods

### 2.1. Study Design and Setting

This cross-sectional survey was conducted between October 2024 and January 2025 in the city of Debrecen, located in Hajdú-Bihar county in Hungary. The study was conducted in accordance with the Declaration of Helsinki. Prior to the commencement of the study, ethical approval was obtained from the Regional and Institutional Research Ethics Committee of the Clinical Center of the University of Debrecen (Reference number: DE RKEB/IKEB No. 6685A-2024). All participants provided written informed consent prior to participating in the study.

### 2.2. Participants and Data Collection

The medical student cohort was surveyed using anonymous, self-administered questionnaires during family medicine seminars. Concurrently, practicing general practitioners were assessed using the exact same instruments during professional continuing medical education (CME) courses. Regarding data quality and processing, only fully completed questionnaires were included in the statistical analysis; incomplete questionnaires were excluded to ensure data integrity. During the data collection period, a total of 236 questionnaires were collected. Of these, 20 incomplete questionnaires were excluded from the analysis to ensure data integrity, leaving 216 fully completed questionnaires valid for inclusion and yielding an overall response rate of 91.5%.

### 2.3. Questionnaire

The study utilized a comprehensive questionnaire designed to assess the demographic profiles, career motivations, and psychological traits of the participants. The instrument was structured into four primary sections: demographic data, perceived advantages and disadvantages of a career in general practice (Table 1), and the shortened Hungarian version of the Cloninger Temperament and Character Inventory (TCI-55). This specific version of the TCI-55 was validated for the Hungarian population and is subdivided into two main components: temperament dimensions and character dimensions [18]. 

### 2.4. The Shortened Hungarian Version of the Cloninger Temperament and Character Inventory (TCI-55)

This section of the questionnaire assesses the temperament and character dimensions of the respondents based on Cloninger’s psychobiological model of personality. According to Cloninger, it is essential to distinguish between temperament, which is heritable and present from birth, and character dimensions, which are learned through environmental influence. Temperament fundamentally determines our automatic emotional responses to stimuli. In contrast, character is defined by an individual’s concepts regarding themselves, others, and the world, characterizing their goals, worldviews, intentions, and attitudes [19].

Cloninger originally identified three temperament dimensions and three character traits, later expanding the former to four. He proposed that the original three temperament traits (Novelty Seeking, Harm Avoidance, Reward Dependence) correlate with levels of the primary neurotransmitters (dopamine, serotonin, and norepinephrine). No specific neurotransmitter was assigned to the fourth dimension, Persistence, as it was separated from Reward Dependence during his later research [19,20].

#### 2.4.1. Definitions of Temperament Traits [21]

Novelty Seeking: Refers to an individual’s exploratory activity, initiative, and the pursuit of potential rewards and new stimuli.
Harm Avoidance: Involves the inhibition of behavior, manifested as caution, tension, shyness, and worrying.
Reward Dependence: Plays a role in the maintenance of behavior, appearing as an increased response to signals of reward.
Persistence: Characterized by determination, perfectionism, ambition, industry, and overachievement.

#### 2.4.2. Definitions of Character Dimensions [21]

Self-Directedness: Indicates the extent to which an individual can accept themselves, find purpose and meaning in life, follow rules to achieve goals, and maintain self-control.
Cooperativeness: Characterized by the acceptance of others, tolerance, helpfulness, agreeableness, and empathy.
Self-Transcendence: Refers to transpersonal identification, acceptance, and spiritual union with nature and the supernatural. It is based on the dissolution of the distinction between the self and others.
Respondents provided their answers using a four-point Likert scale (0: not at all characteristic; 1: rather disagree; 2: rather agree; 3: completely agree).

### 2.5. Statistical Analysis

Before proceeding with the primary statistical analyses, an a priori power analysis was conducted using G*Power software version 3.1.9.7 [22] to determine the minimum required sample size for comparing the means of two independent groups. The parameters were set for a two-tailed test, aiming to detect a medium effect size (Cohen’s d = 0.5) with a significance level of 0.05 and a desired statistical power of 0.80. Anticipating a larger available pool of students compared to practitioners, the allocation ratio (N2/N1) was predefined at 0.5. The analysis indicated that a minimum total sample size of 144 participants (comprising 96 medical students and 48 general practitioners) was required to achieve adequate statistical power (actual power = 0.802). Given that the final recruited sample size of this study (*n* = 216, consisting of 144 students and 72 general practitioners) substantially exceeded this minimum threshold, the study possesses robust statistical power, ensuring the high reliability and validity of the comparative findings.

Statistical analysis was performed using IBM SPSS Statistics version 27. The normality of the data distribution was assessed using the Shapiro–Wilk and Kolmogorov–Smirnov tests, both of which yielded significant results (*p* < 0.05), indicating a non-normal distribution for the temperament and character dimensions. Consequently, the Mann–Whitney U test was employed to compare these dimensions between the groups of general practitioners and medical students. For the analysis of categorical variables, Chi-square tests were conducted; however, in cases where the expected cell count was below 5, Fisher’s exact test was utilized to ensure statistical accuracy. Statistical tests were not performed for variables where any cell count was zero.

## 3. Results

The data collected based on the responses are presented in the following tables. For the statistical analysis of gender, only the “female” and “male” categories were included. Respondents who selected “other” (*n* = 1) or “prefer not to say” (*n* = 3) were excluded from this specific analysis. Including these extremely low frequencies would have violated the assumptions of the statistical tests (such as the Chi-square test) and skewed the results.

Based on the demographic data (Table 1), a significantly higher proportion of general practitioners (49 out of 72) indicated family medicine as their first choice of specialization compared to the students (6 out of 144) (*p* < 0.001). General practitioners had a significantly higher median number of doctors in their families (median = 1) compared to students (median = 0.5) (*p* < 0.001). The number of graduates in the family did not show a statistically significant difference; since both groups shared a median of 5, their averages were reported instead, showing 5.78 for students and 6.62 for general practitioners (*p* = 0.209). The median age of the general practitioner group was 52.5 years, while the student group had a median age of 23 years (*p* < 0.001). There were no statistically significant differences between the two groups regarding gender (*p* = 0.081) or relationship status (*p* = 0.258).

The statistical analysis of the perceived advantages of a career in family medicine revealed significant differences between Hungarian medical students and general practitioners across eleven specific areas (Table 2). In all these instances, a significantly higher proportion of general practitioners identified these aspects as advantages relative to their group size. The most substantial discrepancies were observed in the appreciation of the medical scope of the profession, where “treating a wide variety of diseases” was selected by 42 general practitioners compared to 13 students (*p* < 0.001), and acquiring “broad medical knowledge” was recognized by 32 general practitioners compared to 13 students (*p* < 0.001). Furthermore, “long-term relationships with patients” was chosen by 34 general practitioners and 20 students (*p* < 0.001), while the role “because of preventive medicine” was selected by 25 general practitioners and 17 students (*p* < 0.001). Regarding lifestyle and work structure, “flexible working hours” was selected by 31 general practitioners and 27 students (*p* < 0.001), “family-friendly work” was selected by 27 general practitioners and 28 students (*p* = 0.004), and a “diverse daily routine” was chosen by 21 general practitioners versus 12 students (*p* < 0.001). Additional significant differences were found for “fewer night shifts” (20 general practitioners vs. 20 students; *p* = 0.013), the “opportunity to work in emergency medicine” (15 general practitioners vs. 9 students; *p* = 0.001), “secure income” (13 general practitioners vs. 9 students; *p* = 0.007), and “regular working hours” (10 general practitioners vs. 8 students; *p* = 0.037).

Regarding the perceived disadvantages of the field, the data also demonstrated significant differences between the two groups across seven specific areas (Table 3). Students selected certain negative traits at a significantly higher frequency; the most striking difference was the perception of the field as “boring,” which was chosen by 82 students but only 1 general practitioner (*p* < 0.001). Similarly, dealing with “trivial patient problems” was considered a disadvantage by 44 students compared to 7 general practitioners (*p* = 0.001), and the feeling that the work is “isolating” was selected by 36 students versus 3 general practitioners (*p* < 0.001). Conversely, general practitioners identified specific daily and systemic challenges as disadvantages significantly more often than the student group. “Stress” was selected by 15 general practitioners compared to 4 students (*p* < 0.001), and “media opinion” was marked by 12 general practitioners versus 6 students (*p* = 0.002). Finally, “workload” was chosen by 9 general practitioners and 5 students (*p* = 0.017), and “time constraints” was selected by 8 general practitioners compared to 5 students (*p* = 0.035).

When evaluating the psychological profiles via the TCI-55, two of the main temperament dimensions demonstrated statistically significant differences between the cohorts (Table 4). Significant differences were found in Reward Dependence, where students recorded a higher median score of 5.50 compared to the general practitioners’ median of 4.00 (*p* < 0.001). For Novelty Seeking, both groups shared a median of 4.00, so the difference was reflected in their averages; students scored a higher mean of 4.65 compared to the general practitioners’ mean of 3.54 (*p* < 0.001). Within the temperament subscales, significant differences were explicitly isolated to three areas. For the Novelty Seeking subscale of disorderliness versus regimentation, students had a median of 1.00 compared to the general practitioners’ median of 0.00 (*p* < 0.001). In the Reward Dependence subscales, significant differences were found in attachment versus detachment (student median 1.50 vs. general practitioner median 1.00; *p* = 0.001) and in dependence versus independence (student median 3.00 vs. general practitioner median 1.00; *p* < 0.001). Conversely, no statistically significant differences were found between students and general practitioners across any of the main character dimensions. However, significant differences were identified in three specific character subscales. For the Self-Directedness subscale of enlightened second nature versus conflicting habits, students recorded a median of 1.00 while general practitioners had a median of 0.00 (*p* = 0.035). For the Self-Directedness subscale of self-acceptance versus self-striving, both groups shared a median of 1.00, but students had a higher mean score of 0.82 compared to the general practitioners’ mean of 0.63 (*p* = 0.046). Finally, in the Cooperativeness subscale of compassion versus revengefulness, both groups shared a median of 0.00, with the difference reflected in the students’ mean of 0.28 compared to the general practitioners’ mean of 0.09 (*p* = 0.018).

## 4. Discussion

The primary objective of this study was to investigate the reality of the stereotypes surrounding general practice among medical students and practicing general practitioners (GPs). Furthermore, it aimed to assess the perceived advantages and disadvantages of family medicine within a Hungarian context, outline the temperament and character traits of those drawn to this specialty, and compare these domestic findings with international literature.

When evaluating the demographic data, it is crucial to note that the comparisons were drawn between the overall cohort of medical students and practicing general practitioners. A subgroup analysis between students choosing and those rejecting family medicine was not conducted, as the number of students in our sample who indicated family medicine as their primary choice was extremely low (*n* = 6, representing only 4.2% of the students).

However, this strikingly low proportion is a significant finding in itself: it clearly indicates the current unpopularity of the profession and the widespread lack of interest among students. This trend is not unique to Hungary but is recognized as a global issue in the literature. For instance, in a Greek survey, only 44 out of 1021 medical students (4.3%) selected general practice as a possible career option, primarily due to the perceived lack of specialization and the profession’s low social and medical prestige [23]. Similarly low levels of interest, unpopularity of the specialty, and prevalent negative stereotypes among students have been reported in other international studies [24] as well as in domestic surveys [25]. Attempting to draw conclusions from a subgroup of merely 6 students against the majority of 135 would not have yielded reliable insights, making the examination of the entire student base the most appropriate approach.

Comparing the entire student sample and practicing GPs to previous domestic research reveals several similarities. An earlier Hungarian study indicated that a rural background, female gender, older age, and marital status are not definitive determining factors for medical students in their career choice [25]. Our data largely support this: although a higher proportion of our total respondents were from rural areas compared to the capital, and there were more women and individuals in relationships among the GPs, these differences cannot be considered statistically significant when factoring in the demographic indicators of the general national population. Internationally, studies frequently characterize those choosing family medicine as being older, female, from rural or smaller town backgrounds, and in a relationship [26].

The identified advantages of a career in family medicine align closely with the literature, expanding upon the frequently cited benefits. Previous domestic research highlighted the unique doctor-patient relationship and diverse daily work as primary positives [25]. Based on our respondent data, this list can be expanded to include flexible working hours, fewer night shifts, secure income, a role in preventive medicine, the opportunity to work in emergency medicine, broad medical knowledge, and family-friendly work. Internationally, universally positive aspects of general practice share these sentiments. For instance, a study in Germany found that the desire for diversity, long-term patient relationships, independence, and a good work–life balance were the most common arguments for choosing general practice [27]. Similarly, research from Greece emphasized a guaranteed job opportunity and a good work–life balance as highly valued advantages [23].

Our data shows that students overwhelmingly view family medicine as an “easy choice,” “boring,” “isolating,” and burdened with “trivial patient problems.” These sentiments directly mirror international findings. A survey in Singapore noted that students perceived the work as trivial, repetitive, and characterized by the “low clinical competence” of GPs [24]. Similarly, German graduates deterred from the field cited its monotonous nature and the perception of the GP as a mere “dispatcher” with limited care capabilities [27]. These stereotypes are deeply rooted in the university environment, where education is often highly specialized and hospital-centric, fostering the belief that family medicine lacks clinical ambition.

However, based on the responses from the practicing GPs in our study, there appears to be a stark misalignment between academic perception and clinical reality. The disadvantages identified by the GPs reflect realistic systemic and daily challenges rather than inherent flaws in the medical discipline itself. These include heavy workload, stress, extensive paperwork, time constraints, and negative media opinions. Dealing with trivial patient problems was acknowledged by a small fraction of GPs (9.7%), suggesting that while this perception holds partial truth, it is vastly exaggerated by students. It is important to interpret these findings within the specific context of the Hungarian healthcare system, where post-socialist structural legacies, unique financing models, and a severe workforce shortage create distinct administrative and systemic burdens that differ significantly from practices in the United States or Western Europe. These localized, realistic challenges are nevertheless supported by broader international data, where literature frequently cites heavy workload, lower salaries, immense paperwork, lack of prestige within medical and social circles, and limited career and specialization opportunities as major drawbacks [23,28].

An evaluation of inherited temperament traits revealed highly significant differences, specifically in Novelty Seeking (*p* < 0.001) and Reward Dependence (*p* < 0.001), with the medical student cohort scoring markedly higher in both areas compared to practicing general practitioners. This observation aligns directly with broader international research indicating that medical students typically exhibit elevated levels of Novelty Seeking and Reward Dependence—traits that are generally more pronounced in younger populations and tend to temper naturally with age and life experience [29,30,31]. While the literature suggests that specific temperament profiles may influence a student’s long-term suitability for the unpredictable environment of primary care, our cross-sectional data limits our ability to definitively state that these traits alone dictate career fit.

The significant reductions observed in Self-Acceptance (SD4), Enlightened Second Nature (SD5), and Compassion (CO4) among general practitioners compared to medical students warrant careful interpretation. In the context of Cloninger’s psychobiological model, diminished Self-Directedness is a primary predictor of burnout syndrome and depressive states in healthcare professionals [32]. The decline in Self-Acceptance and Enlightened Second Nature may reflect the systemic realities of modern medical practice; as physicians face compounding administrative burdens and severe time constraints, they frequently experience a profound dissonance between their core medical values and their forced daily habits. This systemic friction can manifest as decreased self-esteem, chronic self-striving, and conflicting habits (low SD4 and SD5), which mirror the developmental trajectory of occupational burnout [33]. However, because our study did not utilize a dedicated burnout assessment tool, we cannot definitively attribute these observed TCI differences to clinical burnout or moral distress, highlighting an area requiring further targeted research.

The observed variations in character traits associated with compassion, self-acceptance, and enlightened second nature among practicing GPs represent a potential public health vulnerability. Burnout and moral distress in primary care do not merely affect the individual physician; the literature extensively documents that they precipitate a cascade of negative population health outcomes, including increased medical errors, reduced patient adherence, and diminished efficacy of preventive care interventions [34]. Furthermore, the pervasive negative stereotypes held by medical students perpetuate a vicious cycle: as fewer graduates enter family medicine, the systemic workload on the aging cohort of existing GPs intensifies, exacerbating systemic strain and inadvertently validating the students’ fears of an overburdened profession. To safeguard public health, structural interventions must go beyond simply increasing general medical school enrollment. Policy frameworks must urgently address the administrative and systemic stressors that erode physician well-being—recognizing that provider health is a fundamental prerequisite for patient health [35]. Simultaneously, medical education must actively dismantle hospital-centric biases by integrating early, high-quality community-based primary care experiences. Ultimately, mitigating systemic stressors and restructuring the specialty’s perception are not just occupational challenges, but urgent public health priorities required to maintain a sustainable, equitable, and proactive healthcare system [36].

While our study originally aimed to quantify and describe the psychological characteristics of students specifically interested in a general practitioner (GP) career, a primary limitation is that evaluating the overall student cohort did not allow us to fully achieve this specific objective. This limitation stems directly from the exceptionally low number of medical students who identified general practice as their first choice of specialization (*n* = 6, representing only 4.2% of the student cohort). This remarkably small sample size prevented a reliable subgroup statistical analysis. Consequently, we could not definitively determine whether students choosing this path possess distinct psychological profiles from their peers at the start of their careers.

Furthermore, while the observed differences in TCI scores between medical students and practicing GPs offer potential insights, these results are influenced by significant demographic and situational factors. The higher levels of Novelty Seeking and Reward Dependence in students are traits generally more pronounced in younger populations and tend to decrease naturally with age and life experience. Therefore, the temperament gap may be heavily influenced by the median age difference (23 years vs. 52.5 years) rather than career choice alone. Finally, while the character profile of the practicing GPs—specifically the significant reductions in Self-Acceptance, Enlightened Second Nature, and Compassion—aligns with patterns often seen in occupational burnout and moral distress, this study did not utilize a dedicated burnout assessment tool. Therefore, we cannot establish a direct causal relationship, and the degree to which these psychological shifts result from systemic underfunding and administrative burdens remains a hypothesis. Future research should prioritize measuring burnout directly to better understand how the deteriorating mental well-being of active GPs might deter medical students.

## 5. Conclusions

Hungary’s primary healthcare system is facing a critical bottleneck driven by a severe shortage of General Practitioners. This challenge is further compounded by the profession’s stark unpopularity among the next generation of physicians, as evidenced by the alarmingly low percentage of medical students who indicate family medicine as their primary career choice. A major driver of this lack of interest appears to stem from deeply rooted stereotypes held by university students, who frequently perceive general practice as an “easy choice” that is “boring,” “isolating,” and burdened with “trivial patient problems.” In contrast, practicing GPs report a vastly different reality, identifying heavy workloads, stress, severe time constraints, and extensive administrative paperwork as their primary occupational challenges.

Psychological assessments using the TCI-55 further highlight this generational and occupational divide, with medical students naturally exhibiting significantly higher levels of inherited temperament traits like Novelty Seeking and Reward Dependence compared to practicing physicians. Additionally, practicing GPs demonstrate lower scores in character dimensions such as Self-Acceptance, Enlightened Second Nature, and Compassion. While within Cloninger’s psychobiological model this specific character profile is often associated with occupational burnout and systemic moral distress, our study did not directly measure burnout; therefore, direct causal relationships cannot be definitively established. Nevertheless, these findings strongly suggest an urgent need to address the structural and administrative realities of primary care, both to support the well-being of current practitioners and to ensure the sustainable recruitment of future physicians.

## Figures and Tables

**Table 1:** Demographic data.

Variable	Total (*n* = 216)	Hungarian Student (*n* = 144)	General Practitioner (*n* = 72)	*p*-Value (Chi-Square)
Gender				0.081
Female	136 (63.0%)	96 (66.7%)	40 (55.6%)	
Male	76 (35.2%)	44 (30.6%)	32 (44.4%)	
Other	1 (0.5%)	1 (0.7%)	0 (0.0%)	
Prefer not to say	3 (1.4%)	3 (2.1%)	0 (0.0%)	
Relationship status				0.258
Single	59 (27.3%)	43 (29.9%)	16 (22.2%)	
Not single	156 (72.2%)	101 (70.1%)	55 (76.4%)	
Place of residence				0.228
Capital city	10 (4.6%)	4 (2.8%)	6 (8.3%)	
City with county rights	104 (48.1%)	75 (52.1%)	29 (40.3%)	
Town/City	68 (31.5%)	42 (29.2%)	26 (36.1%)	
Large village	4 (1.9%)	3 (2.1%)	1 (1.4%)	
Village	29 (13.4%)	19 (13.2%)	10 (13.9%)	
First choice of specialization				<0.001
Family medicine	55 (25.5%)	6 (4.2%)	49 (68.1%)	
Other	157 (72.7%)	135 (93.8%)	22 (30.6%)	
Average and Median age	-	23.99 (M = 23)	52.77 (M = 52.5)	<0.001
Average and Median number of graduates in family	-	5.78 (M = 5)	6.62 (M = 5)	0.209
Average and Median number of doctors in family	-	1.22 (M = 0.5)	2.87 (M = 1)	<0.001

**Table 2:** Advantages of a career in family medicine.

Variable	Group	Not Selected *n* (%)	Selected *n* (%)	*p*-Value
Secure job	Hungarian student	134 (93.1%)	10 (6.9%)	0.097
	General Practitioner	62 (86.1%)	10 (13.9%)	
Future prospects in the job	Hungarian student	138 (95.8%)	6 (4.2%)	0.208
	General Practitioner	66 (91.7%)	6 (8.3%)	
Flexible working hours	Hungarian student	117 (81.3%)	27 (18.8%)	<0.001
	General Practitioner	41 (56.9%)	31 (43.1%)	
Regular working hours	Hungarian student	136 (94.4%)	8 (5.6%)	0.037
	General Practitioner	62 (86.1%)	10 (13.9%)	
Diverse daily routine	Hungarian student	132 (91.7%)	12 (8.3%)	<0.001
	General Practitioner	51 (70.8%)	21 (29.2%)	
Fewer night shifts	Hungarian student	124 (86.1%)	20 (13.9%)	0.013
	General Practitioner	52 (72.2%)	20 (27.8%)	
Good salary	Hungarian student	133 (92.4%)	11 (7.6%)	0.396
	General Practitioner	64 (88.9%)	8 (11.1%)	
Secure income	Hungarian student	135 (93.8%)	9 (6.3%)	0.007
	General Practitioner	59 (81.9%)	13 (18.1%)	
Participation in R&D	Hungarian student	143 (99.3%)	1 (0.7%)	0.573
	General Practitioner	71 (98.6%)	1 (1.4%)	
Treating a wide variety of diseases	Hungarian student	131 (91.0%)	13 (9.0%)	<0.001
	General Practitioner	30 (41.7%)	42 (58.3%)	
Staying up to date with research	Hungarian student	143 (99.3%)	1 (0.7%)	0.109
	General Practitioner	69 (95.8%)	3 (4.2%)	
Because of preventive medicine	Hungarian student	127 (88.2%)	17 (11.8%)	<0.001
	General Practitioner	47 (65.3%)	25 (34.7%)	
Low physical stress	Hungarian student	136 (94.4%)	8 (5.6%)	1.000
	General Practitioner	68 (94.4%)	4 (5.6%)	
Low mental stress	Hungarian student	134 (93.1%)	10 (6.9%)	0.345
	General Practitioner	70 (97.2%)	2 (2.8%)	
Long-term relationships with patients	Hungarian student	124 (86.1%)	20 (13.9%)	<0.001
	General Practitioner	38 (52.8%)	34 (47.2%)	
Opportunity to work in emergency medicine	Hungarian student	135 (93.8%)	9 (6.3%)	0.001
	General Practitioner	57 (79.2%)	15 (20.8%)	
Broad medical knowledge	Hungarian student	131 (91.0%)	13 (9.0%)	<0.001
	General Practitioner	40 (55.6%)	32 (44.4%)	
Plenty of free time	Hungarian student	131 (91.0%)	13 (9.0%)	0.868
	General Practitioner	65 (90.3%)	7 (9.7%)	
Separation of professional & private life	Hungarian student	136 (94.4%)	8 (5.6%)	0.256
	General Practitioner	65 (90.3%)	7 (9.7%)	
Ability to work part-time	Hungarian student	133 (92.4%)	11 (7.6%)	0.228
	General Practitioner	70 (97.2%)	2 (2.8%)	
Family-friendly work	Hungarian student	116 (80.6%)	28 (19.4%)	0.004
	General Practitioner	45 (62.5%)	27 (37.5%)	
Publicly recognized	Hungarian student	144 (100.0%)	0 (0.0%)	-
	General Practitioner	69 (95.8%)	3 (4.2%)	
Career goals	Hungarian student	144 (100.0%)	0 (0.0%)	-
	General Practitioner	72 (100.0%)	0 (0.0%)	
Personal goals	Hungarian student	136 (94.4%)	8 (5.6%)	0.662
	General Practitioner	69 (95.8%)	3 (4.2%)	
Positive reputation within medicine	Hungarian student	143 (99.3%)	1 (0.7%)	-
	General Practitioner	72 (100.0%)	0 (0.0%)	
Positive reputation in the media	Hungarian student	143 (99.3%)	1 (0.7%)	-
	General Practitioner	72 (100.0%)	0 (0.0%)	
Priority in medical training	Hungarian student	143 (99.3%)	1 (0.7%)	0.258
	General Practitioner	70 (97.2%)	2 (2.8%)	

Note: Selected indicates that the respondent actively chose this attribute as an advantage of the general practitioner career. Not selected indicates that the respondent did not choose this attribute. Percentages are calculated based on the total number of respondents within each specific group (Hungarian Students *n* = 144; General Practitioners *n* = 72).

**Table 3:** Disadvantages of a career in family medicine.

Variable	Group	Not Selected *n* (%)	Selected *n* (%)	*p*-Value
Workload	Hungarian student	139 (96.5%)	5 (3.5%)	0.017
	General Practitioner	63 (87.5%)	9 (12.5%)	
Easy choice	Hungarian student	113 (78.5%)	31 (21.5%)	-
	General Practitioner	72 (100.0%)	0 (0.0%)	
Boring	Hungarian student	62 (43.1%)	82 (56.9%)	<0.001
	General Practitioner	71 (98.6%)	1 (1.4%)	
Stress	Hungarian student	140 (97.2%)	4 (2.8%)	<0.001
	General Practitioner	57 (79.2%)	15 (20.8%)	
Negative things are said about it	Hungarian student	134 (93.1%)	10 (6.9%)	1.000
	General Practitioner	67 (93.1%)	5 (6.9%)	
Paperwork	Hungarian student	107 (74.3%)	37 (25.7%)	0.739
	General Practitioner	55 (76.4%)	17 (23.6%)	
Trivial patient problems	Hungarian student	100 (69.4%)	44 (30.6%)	0.001
	General Practitioner	65 (90.3%)	7 (9.7%)	
Time constraints	Hungarian student	139 (96.5%)	5 (3.5%)	0.035
	General Practitioner	64 (88.9%)	8 (11.1%)	
Complaints	Hungarian student	134 (93.1%)	10 (6.9%)	0.345
	General Practitioner	70 (97.2%)	2 (2.8%)	
I think it’s for those who can’t do anything else	Hungarian student	135 (93.8%)	9 (6.3%)	0.529
	General Practitioner	69 (95.8%)	3 (4.2%)	
Media opinion	Hungarian student	138 (95.8%)	6 (4.2%)	0.002
	General Practitioner	60 (83.3%)	12 (16.7%)	
Isolating	Hungarian student	108 (75.0%)	36 (25.0%)	<0.001
	General Practitioner	69 (95.8%)	3 (4.2%)	
Uncertainty	Hungarian student	124 (86.1%)	20 (13.9%)	1.000
	General Practitioner	62 (86.1%)	10 (13.9%)	

Note: Selected indicates that the respondent actively chose this attribute as an advantage of the general practitioner career. Not selected indicates that the respondent did not choose this attribute. Percentages are calculated based on the total number of respondents within each specific group (Hungarian Students *n* = 144; General Practitioners *n* = 72).

**Table 4:** Temperament and Character profiles of the groups.

	Hungarian Student		General Practitioner		*p*
	Mean	Median	Mean	Median	
Temperament dimensions					
Novelty Seeking (NS)	4.65	4.00	3.54	4.00	<0.001
NS exploratory excitability–stoic rigidity	1.10	1.00	1.11	1.00	0.731
NS impulsiveness–reflection	1.72	2.00	1.54	2.00	0.124
NS extravagance–reserve	0.92	1.00	0.89	1.00	0.609
NS disorderliness–regimentation	0.90	1.00	0.40	0.00	<0.001
Harm Avoidance (HA)	3.93	4.00	3.91	4.00	0.840
HA anticipatory worry/pessimism–uninhibited optimism	1.51	2.00	1.63	2.00	0.099
HA fear of uncertainty–confidence	0.82	1.00	0.63	0.00	0.109
HA shyness with strangers–gregariousness	0.83	1.00	0.83	1.00	0.895
HA fatigability and asthenia–vigor	0.77	1.00	0.83	1.00	0.479
Reward Dependence (RD)	5.51	5.50	3.91	4.00	<0.001
RD sentimentality–insensitivity	1.35	1.00	1.50	2.00	0.256
RD attachment–detachment	1.73	1.50	1.24	1.00	0.001
RD dependence–independence	2.43	3.00	1.17	1.00	<0.001
Persistence–irresolution (PS)	0.60	1.00	0.57	1.00	0.769
Character dimensions					
Self-Directedness (SD)	2.28	2.00	2.33	2.00	0.885
SD responsibility–blaming	0.26	0.00	0.26	0.00	0.817
SD purposefulness–lack of goal direction	0.20	0.00	0.10	0.00	0.106
SD resourcefulness–inertia	0.28	0.00	0.27	0.00	0.705
SD self-acceptance–self-striving	0.82	1.00	0.63	1.00	0.046
SD enlightened second nature–conflicting habits	0.72	1.00	0.50	0.00	0.035
Cooperativeness (CO)	5.28	5.00	5.14	5.00	0.492
CO social acceptance–social intolerance	0.38	0.00	0.43	0.00	0.572
CO empathy–social disinterest	1.10	1.00	1.06	1.00	0.501
CO helpfulness–unhelpfulness	1.84	2.00	1.84	2.00	0.925
CO compassion–revengefulness	0.28	0.00	0.09	0.00	0.018
CO pure-hearted conscience–self-advantage	1.68	2.00	1.73	2.00	0.545
Self-Transcendence (ST)	2.32	2.00	2.04	1.00	0.254
ST self-forgetfulness–self-conscious experience	0.81	1.00	0.76	1.00	0.770
ST transpersonal identification–self-isolation	0.58	0.00	0.47	0.00	0.256
ST spiritual acceptance–rational materialism	0.93	1.00	0.81	1.00	0.379

## Data Availability

The data presented in this study are available on request from the corresponding author. The data are not publicly available due to ethical reasons.
